# Surgical outcomes following neoadjuvant chemotherapy with and without immunotherapy in patients with triple-negative breast cancer

**DOI:** 10.1007/s12672-024-01349-7

**Published:** 2024-09-20

**Authors:** Anouchka Coste Holt, Courtney A. Smith, Maurice J. Berkowitz, Jennifer L. Baker, Nicholas P. McAndrew, Nimmi S. Kapoor

**Affiliations:** 1 Holy Cross and University of Miami, Fort Lauderdale, FL USA; 2 Division of Surgical Oncology, Department of Surgery, University of California Los Angeles, Los Angeles, CA USA; 3 Division of Hematology and Oncology, Department of Medicine, University of California Los Angeles, Los Angeles, CA USA

**Keywords:** Neoadjuvant immunotherapy, TNBC, Surgical outcomes

## Abstract

**Purpose:**

Adding pembrolizumab to neoadjuvant chemotherapy (NAC) for triple-negative breast cancer (TNBC) improves pathologic complete response (pCR) rates and event-free survival. The impact of adding immunotherapy to NAC on surgical outcomes is unknown. This study compares 90-day post-surgical complications (PSCs) and time to adjuvant treatment among patients undergoing NAC for TNBC with and without immunotherapy.

**Methods:**

Patients treated with NAC alone or with immunotherapy (NAC-I) for stage I–III TNBC between 2018 and 2022 were retrospectively identified at a single academic institution. Kruskal–Wallis rank sum and Fisher's exact tests compared patient sociodemographic and clinical characteristics. Multivariable logistic regression determined odds ratios (OR) predicting PSCs.

**Results:**

Of 54 patients, 29 received NAC alone and 25 received NAC-I. Compared to NAC patients, NAC-I patients had more advanced stage tumors (p = 0.038), and had slightly higher rates of mastectomy with reconstruction (p = 0.193). 72.0% of NAC-I patients experienced a pCR, compared with 44.8% of NAC patients (p = 0.193). There were 10 PSCs (34.5%) in NAC patients compared to 9 PSCs (36.0%) in NAC-I patients (p > 0.99). Regression analysis demonstrated no association of PSCs with NAC-I (OR 0.83, 95% CI 0.19–3.60). Time to adjuvant therapy was shorter for NAC-I patients (28 days vs 36 days, p = 0.013).

**Conclusions:**

Patients with TNBC receiving NAC-I have higher pCR rates and do not appear to have added 90-day PSCs or delays to adjuvant therapy despite trending toward more extensive surgical procedures compared to NAC alone. Larger studies are needed to further evaluate the surgical safety of immunotherapy.

## Introduction

Triple-negative breast cancer (TNBC) accounts for 10–20% of all breast cancers and treatment with neoadjuvant chemotherapy has become standard practice for this more aggressive form of breast cancer. Neoadjuvant chemotherapy regimens for TNBC have consisted of anthracyclines, taxanes, and platinums and are able to achieve a pathologic complete response (pCR) rate of up to 40–50% [1]. Patients who achieve a pCR have an improved prognosis and disease-free survival compared to those who do not have a pCR, and those with residual disease have improved survival when given additional post-neoadjuvant therapy [2–4]. The recent addition of immunotherapy in the form of immune checkpoint inhibitors to neoadjuvant breast cancer treatment regimens has transformed management and prognosis for patients with TNBC. Pembrolizumab is an anti–programmed death 1 (PD-1) monoclonal antibody that was first shown to improve pCR rate in high-risk, early-stage TNBC with low-grade toxic effects in patients demonstrating positive correlation with tumor PD-L1 expression when used as first-line treatment [5]. Subsequently, pembrolizumab was also shown to improve pCR rates when given with NAC for stage II-III breast cancer in both I-SPY2 and KEYNOTE-522 regardless of PDL-1 expression [6–8]. Moreover, KEYNOTE-522 demonstrated a 3-year estimated disease-free survival improvement from 76.8 to 84.5% in the control group versus pembrolizumab, leading to FDA approval of the addition of pembrolizumab to the neoadjuvant treatment of stage II–III TNBC in 2021[9].

The inclusion of pembrolizumab, however, is not without consequences, and severe side effects of febrile neutropenia, anemia, and pyrexia are reported in over 30% who take this drug, while less common (but oftentimes permanent) immune-based side effects of thyroiditis, pneumonitis, and adrenal insufficiency are also well-established risks [9, 10]. Limited data is available to demonstrate how treatment-related side effects and the receipt of neoadjuvant pembrolizumab translate to surgical complications. The potential added risk of immunotherapy to surgical outcomes is especially important when considering the younger population of TNBC patients who may desire reconstruction.

Furthermore, surgical complications may lead to further delays in adjuvant systemic therapy and radiation. In a recent retrospective study of a single cohort of patients with stage I–III TNBC treated with NAC and pembrolizumab, 24.1% of patients had perioperative complications, including delays in surgical care, alterations in their surgical plan, and postoperative complications [11]. However, conclusions regarding the influence of immunotherapy on complication rates are limited since there was no comparison group [11]. The current study aims to compare the odds of developing 90-day post-surgical complications (PSCs) and delays in subsequent adjuvant therapy among patients undergoing NAC for TNBC breast cancer with and without pembrolizumab.

## Materials and methods

All patients with stage I–III TNBC who had surgery at University of California Los Angeles (UCLA) between 2018–2022 and were treated with neoadjuvant chemotherapy either alone or with immunotherapy (NAC vs NAC-I, respectively) were retrospectively identified. Stage IV patients were excluded from the study. Data was extracted electronically using predefined inclusion criteria to yield information regarding patient sociodemographic and clinical characteristics. This retrospective study was approved by the UCLA Human Research Committee Institutional Review Board (Project No: 23–000376) and performed in line with the principles of the Declaration of Helsinki. In this study, the informed consent requirement has been waived by the Human Research Committee Institutional Review Board of UCLA.

### Study variables and outcomes of interest

In this exploratory study, primary outcome of interest was rate of any surgical complication within 90 days. Secondary endpoint was difference in complication rate between patients receiving NAC vs NAC-I. Post-surgical complications were defined as clinical deviations from the normal postoperative course occurring within 90 days of surgery and included major complications defined as flap necrosis or bleeding requiring re-operation, or hospitalization. Minor complications were those not requiring reoperation or hospitalization and included seroma undergoing aspiration, cellulitis requiring antibiotics, and wound complications undergoing in-office debridement. The type of surgical procedures performed were determined from operative notes. The type of nodal staging was defined as patients having sentinel lymph node biopsy, including targeted dissections (SLNB) or axillary lymph node dissection (ALND). If targeted dissection was converted to completion axillary dissection, then this was counted as an ALND. Breast surgery was defined as mastectomy or lumpectomy, and reconstruction, including expander-based or autologous tissue-based, was classified as performed or not performed. The interval from surgery to complication was defined as the number of days between the date of surgery and the date of the first post-surgical complication.

Information on immunohistochemical receptors (ER, PR, and HER2), FISH (HER2neu), the presence of genetic mutations, and anatomic staging were obtained from post-surgical final pathology reports. Pathological complete response (pCR) was defined by final pathology as the absence of residual invasive cancer in breast and nodal tissue (ypT0/is ypN0).

Neoadjuvant therapy was defined as any systemic therapy given before surgery including immunotherapy and/or chemotherapy. Immunotherapy consisted of pembrolizumab. Patients receiving pembrolizumab also received chemotherapy, however specific regimens varied between KEYNOTE regimen^7^ and abbreviated regimens. Chemotherapy regimens in both groups varied and included a combination of agents including taxane, cyclophosphamide, adriamycin, and carboplatin.

An additional secondary endpoint was time to adjuvant therapy. Adjuvant therapy was defined as immunotherapy, chemotherapy, or radiation after surgical intervention. The interval from NAC to surgery was defined as the number of days between the final neoadjuvant chemotherapy dosage and the surgical date. The interval from surgery to adjuvant treatment was defined as the number of days between the date of first breast oncologic surgery and the date of first adjuvant therapy.

Additional clinical parameters included menopausal status, current pregnancy at the time of diagnosis, and type II diabetes. Demographic parameters included are age at diagnosis, sex, race, ethnicity, smoking history, and body mass index (BMI).

### Statistical analysis

Data cleaning, variable creation, and statistical analysis were performed in R 4.3.0 (https://www.r-project.org/), and an alpha of p < 0.05 was chosen for all analyses. Clinical characteristics and demographics were summarized using the Kruskal–Wallis rank sum and Fisher's exact tests, as appropriate. Missing data for a given variable were excluded from any data analysis including that particular variable. Multivariable regression results are presented as adjusted odds ratios (aOR) of post-surgical complications or PSCs. Covariates included in regression analysis evaluating factors associated with PSC were factors known to be associated with surgical complications including age, diabetes, smoking, BMI and extent of surgery including both breast and axillary surgery and reconstruction. Variables that were significant on univariate analysis were also included in the regression analysis. Model collinearity was absent, as determined by variance inflation factors less than 5. Multiple comparison correction was performed for all p-values using the Benjamini–Hochberg method with a false discovery rate of 0.05.

## Results

A total of 54 patients were identified, with 29 receiving NAC alone and 25 receiving NAC with immunotherapy (NAC-I). All patients were female and Table 1 shows patient characteristics of the study population. Between the two groups, there was no significant difference in age, menopausal status, smoking history, BMI, ethnicity, race, presence of genetic mutation, or history of recent pregnancy on univariate analysis. The most common pathogenic mutations were in BRCA1 and BRCA2 genes (n = 7 and n = 4, respectively). A higher proportion of patients receiving NAC-I were diagnosed with stage II (56%, n = 14) or III (32%, n = 8) TNBC compared to patients in the NAC group, which was comprised of 48.3% stage II (n = 14) and 3.4% stage III (n = 1) tumors (p = 0.038). Duration of time from NAC-I or NAC to surgery was similar between groups at median 31 days (range 4–77) for NAC-I and 34 days (range 23–51) for NAC (p = 0.12).

**Table 1:** Comparison of patients with TNBC who received NAC vs NAC-I

		Neoadjuvant type		p-value
Characteristic	Overall, N = 54	NAC N = 29	NAC-I N = 25	
Age in years, Median (range)	50 (29—84)	57 (33—84)	47 (29—79)	0.166
Race, n (%)				> 0.99
White	n = 34 (63.0%)	n = 19 (65.5%)	n = 15 (60.0%)	
Non-White	n = 15 (27.8%)	n = 8 (27.6%)	n = 7 (28.0%)	
Unknown	n = 5 (9.3%)	n = 2 (6.9%)	n = 3 (12.0%)	
Ethnicity, n (%)				> 0.99
Not Hispanic or Latino	n = 43 (81.1%)	n = 25 (86.2%)	n = 18 (75.0%)	
Hispanic or Latino	n = 8 (15.1%)	n = 3 (10.3%)	n = 5 (20.8%)	
Chose not to answer	n = 2 (3.8%)	n = 1 (3.4%)	n = 1 (4.2%)	
NA	1	0	1	
BMI (kg/m^2^), n (%)				> 0.99
< 30	n = 44 (81.5%)	n = 23 (79.3%)	n = 21 (84.0%)	
≥ 30	n = 10 (18.5%)	n = 6 (20.7%)	n = 4 (16.0%)	
Menopausal status, n (%)				0.139
Premenopausal	n = 29 (53.7%)	n = 11 (37.9%)	n = 18 (72.0%)	
Postmenopausal	n = 25 (46.3%)	n = 18 (62.1%)	n = 7 (28.0%)	
Pregnant, n (%)				> 0.99
No	n = 51 (94.4%)	n = 28 (96.6%)	n = 23 (92.0%)	
Yes	n = 3 (5.6%)	n = 1 (3.4%)	n = 2 (8.0%)	
Type II diabetes, n (%)				> 0.99
No	n = 48 (88.9%)	n = 25 (86.2%)	n = 23 (92.0%)	
Yes	n = 6 (11.1%)	n = 4 (13.8%)	n = 2 (8.0%)	
Smoker, n (%)				> 0.99
No	n = 52 (96.3%)	n = 28 (96.6%)	n = 24 (96.0%)	
Yes	n = 2 (3.7%)	n = 1 (3.4%)	n = 1 (4.0%)	
Pathogenic mutation, n (%)				0.823
Positive	n = 36 (66.7%)	n = 21 (72.4%)	n = 15 (60.0%)	
Negative	n = 18 (33.3%)	n = 8 (27.6%)	n = 10 (40.0%)	
Anatomic stage, n (%)				**0.038**
I	17 (31.5%)	14 (48.3%)	3 (12.0%)	
II	28 (51.9%)	14 (48.3%)	14 (56.0%)	
III	9 (16.7%)	1 (3.4%)	8 (32.0%)	
Pathologic N category, n (%)				> 0.99
1–3	6 (11.1%)	3 (10.3%)	3 (12.0%)	
x–0	48 (88.9%)	26 (89.7%)	22 (88.0%)	
Pathologic T category, n (%)				0.333
0	29 (53.7%)	12 (41.4%)	17 (68.0%)	
1–2	22 (40.7%)	15 (51.7%)	7 (28.0%)	
is	3 (5.6%)	2 (6.9%)	1 (4.0%)	
Clinical N category, n (%)				0.139
0	38 (70.4%)	25 (86.2%)	13 (52.0%)	
1	10 (18.5%)	3 (10.3%)	7 (28.0%)	
2–3	6 (11.1%)	1 (3.4%)	5 (20.0%)	
Clinical T category, n (%)				
1	19 (35.2%)	13 (44.8%)	6 (24.0%)	
2	28 (51.9%)	14 (48.3%)	14 (56.0%)	
3	6 (11.1%)	2 (6.9%)	4 (16.0%)	
Breast surgery, n (%)				0.299
Lumpectomy	24 (44.4%)	16 (55.2%)	8 (32.0%)	
Mastectomy	30 (55.6%)	13 (44.8%)	17 (68.0%)	
Reconstruction, n (%)				0.193
No	25 (46.3%)	17 (58.6%)	8 (32.0%)	
Yes	29 (53.7%)	12 (41.4%)	17 (68.0%)	
Type of reconstruction, n (%)				> 0.99
DIEP flap	7 (24.1%)	3 (25.0%)	4 (23.5%)	
Tissue expander	22 (75.9%)	9 (75.0%)	13 (76.5%)	
Nodal sampling, n (%)				> 0.99
Axillary lymph node dissection	5 (9.3%)	2 (6.9%)	3 (12.0%)	
Sentinel lymph node biopsy/ targeted axillary dissection	49 (90.7%)	27 (93.1%)	22 (88.0%)	
Surgical complications, n (%)				> 0.99
No	35 (64.8%)	19 (65.5%)	16 (64.0%)	
Yes	19 (35.2%)	10 (34.5%)	9 (36.0%)	
pCR, n (%)				0.193
No	23 (42.6%)	16 (55.2%)	7 (28.0%)	
Yes	31 (57.4%)	13 (44.8%)	18 (72.0%)	

*BMI*  body mass index, *NAC*  neoadjuvant chemotherapy, *NAC-I*  neoadjuvant chemotherapy and immunotherapy

### Surgical management, pCR, and PSCs

Over half of patients underwent mastectomy (n = 30, 55.6%), including 28 patients who also had contralateral prophylactic mastectomy and 2 who had unilateral mastectomy, one in each group. Although not statistically significant, more patients were treated with mastectomy in NAC-I group compared to NAC group (68.0% vs 41.8%, p = 0.061). All patients in NAC-I group who underwent mastectomy (n = 17) underwent reconstruction including tissue expander (n = 13, 76.5%) and autologous reconstruction (n = 4, 23.5%) with similar reconstructive rates seen in the NAC group (Table 1). All patients in both groups underwent axillary staging surgery. Only five patients underwent ALND including 2 (6.9%) in the NAC group and 3 (12%) in the NAC-I group. The remaining 49 patients had SLNB with or without targeted dissection, including 93.1% of the NAC group (n = 27) and 88% of the NAC-I group (n = 22, p = 0.65). More patients in the NAC-I group experienced a pCR at 72.0% (n = 18) compared to 44.8% (n = 13) in the NAC group (p = 0.057).

**Fig. 1:**
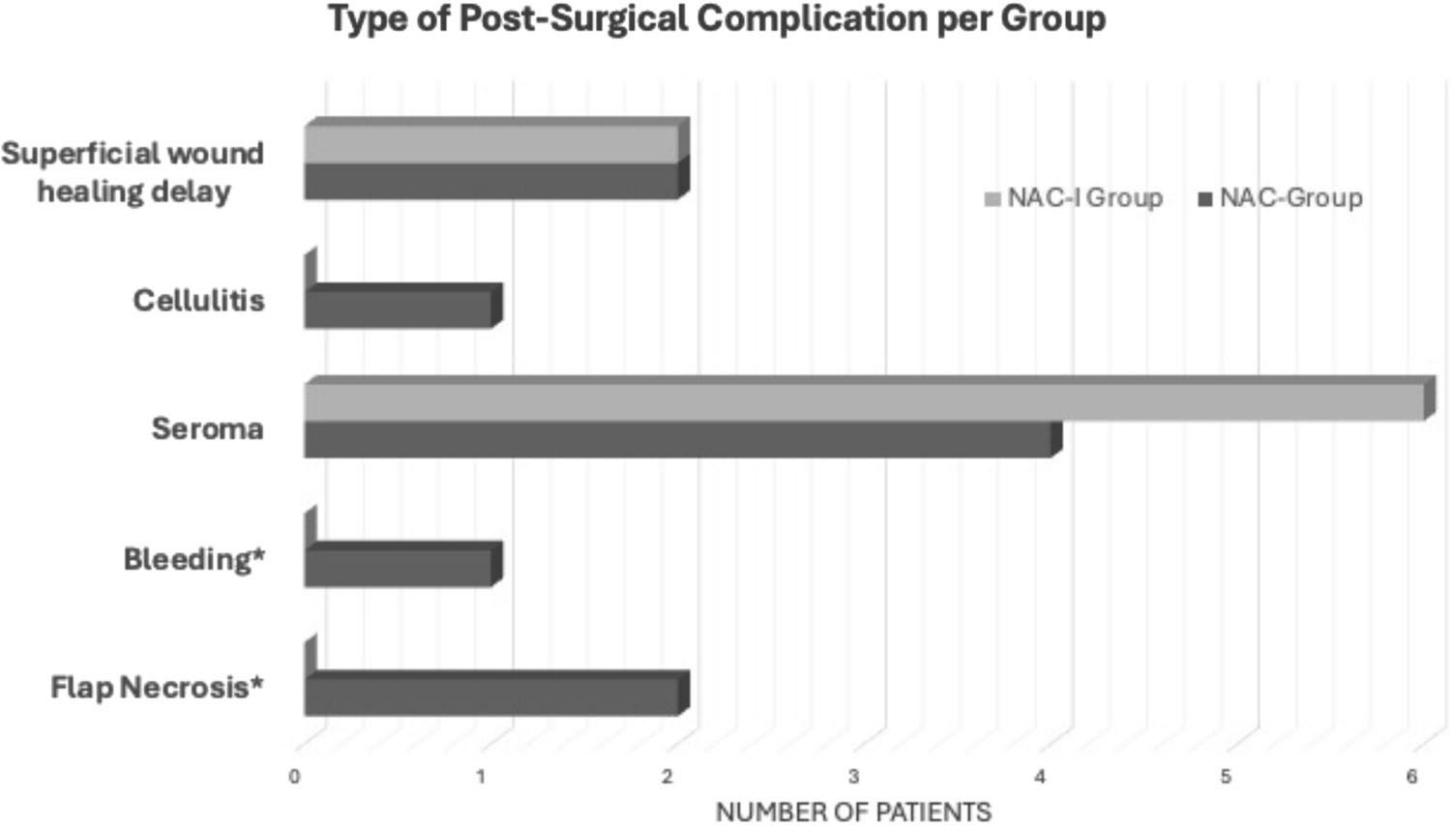
Type of post-surgical complications. *NAC*  neoadjuvant chemotherapy, *NAC-I*  neoadjuvant chemotherapy and immunotherapy. *Denotes major complications

**Fig. 2:**
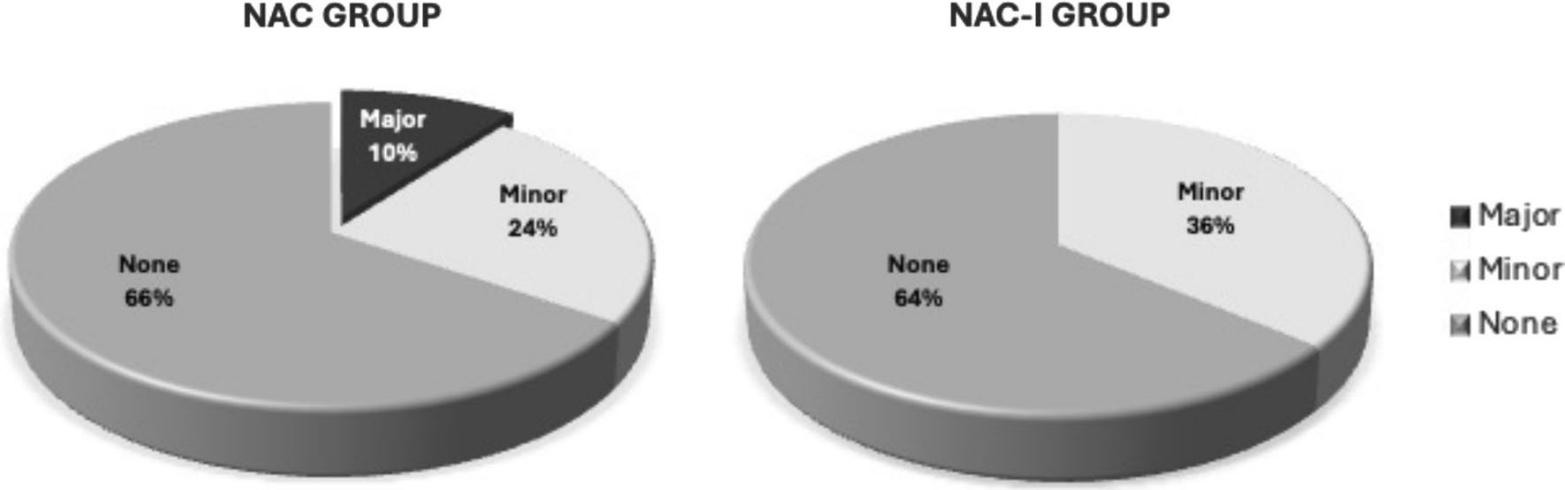
Severity of post-surgical complications. *NAC*  neoadjuvant chemotherapy, *NAC-I*  neoadjuvant chemotherapy and immunotherapy

A total of 19 patients (35.2%) had PSCs (Fig. 1), with no between-group differences (p > 0.99). Median time from surgery to PSC was 15 days and there were more delayed PSCs in NAC-I group at median time of 28 days (range 2–59 days) compared to median 8.5 days (range 1–88 days) in NAC group (p = 0.034). Within the NAC-I group, 9 patients (36.0%) had minor PSCs, which included seroma requiring aspiration (n = 6), cellulitis requiring antibiotics (n = 1), and wound dehiscence requiring local wound care (n = 2). There were no major complications in the NAC-I group. In the NAC group, 10 patients (34.4%) experienced minor (n = 7) or major PSCs (n = 3), which included bleeding or flap necrosis (Fig. 2). Multivariable logistic regression analysis adjusted for covariates of known factors associated with PSC including age, BMI, diabetes, smoking status, and extent surgery (breast surgery, axillary surgery, and reconstruction), controlling for stage, and exploring receipt of immunotherapy demonstrated no association of PSCs with NAC-I (OR 0.83, 95% CI 0.19–3.60, p = 0.926) (Table 2).

**Table 2:** Multivariable logistic regression of 90-day post-surgical complications

Variable	aOR (95% CI)	p-value
Age in years	0.96 (0.92 to 1.01)	0.721
BMI (kg/m^2^)		
< 25	–	
25–30	0.82 (0.13 to 5.02)	0.926
> 30	0.75 (0.18 to 3.18)	0.926
Type II diabetes		
Yes	–	
No	0.31 (0.03 to 3.62)	0.926
Anatomic stage		
II-III	–	
I	0.94 (0.21 to 4.34)	0.940
Smoking		
Never	–	
Current or former	1.18 (0.28 to 4.88)	0.926
Neoadjuvant therapy		
NAC	–	
NACI	0.83 (0.19 to 3.60)	0.926
Axillary staging surgery		
SLNBx	–	
ALND or TAD ± SNLBx	0.57 (0.10 to 3.72)	0.926
Type of surgery		
Lumpectomy	–	
Mastectomy	0.47 (0.07 to 3.32)	0.926
Reconstruction		
Yes	–	
No	0.20 (0.02 to 1.73)	0.721

*BMI*  Body mass index, *NAC*  neoadjuvant chemotherapy, *NACI*  neoadjuvant chemotherapy and immunotherapy, *SLNBx*  Sentinel lymph node biopsy, *ALND*  axillary lymph node dissection, *TAD*  targeted axillary dissection

### Timing from surgery to adjuvant therapy

Adjuvant systemic therapy was given to 59.3% of patients (n = 32) including 21 NAC-I patients (84.0%) and 11 NAC patients (37.9%), p = 0.002. Patients in the NAC-I group were significantly more likely to receive adjuvant immunotherapy whereas patients in NAC group were more likely to receive adjuvant chemotherapy (Table 3). The median duration of time from surgery to first additional adjuvant therapy was less in the NAC-I compared to NAC group (28 vs. 36 days, respectively, p = 0.013).

**Table 3:** Receipt and timing of adjuvant therapy

		Neoadjuvant type		p-value
Characteristic	Overall, n = 54	NAC, n = 29	NACI, n = 25	
Adjuvant radiation	34 (63.0%)	20 (69.0%)	14 (56.0%)	0.4
Adjuvant systemic therapy	32 (59.3%)	11 (37.9%)	21 (84.0%)	**0.002**
Type of systemic therapy				** < 0.001**
Immunotherapy	19 (35.2%)	1 (3.4%)	18 (72.0%)	
Chemotherapy	13 (24.1%)	10 (34.5%)	3 (12.0%)	
None	22 (40.7%)	18 (62.1%)	4 (16.0%)	
Days from surgery to adjuvant therapy, median (range)	33.0 (2.0–123.0)	36.0 (13.0–123.0)	28.0 (2.0–59.0)	**0.013**

*NAC*  neoadjuvant chemotherapy, *NACI*  neoadjuvant chemotherapy and immunotherapy

## Discussion

When added to neoadjuvant chemotherapy, immunotherapy in the form of pembrolizumab significantly improves both pCR rates and event free survival [7, 9]. As predicted by KEYNOTE-522 study, we also observed a significantly higher rate of pCR in the NAC-I group than in the NAC group at 72% vs 44.8%, respectively. At the same time, immunotherapy has been associated with significant side effects [7, 12], but in our cohort, while some patients in both groups did experience delay to surgery after chemotherapy, there was no significant difference in time to surgery after neoadjuvant treatment between the two groups, with a median time to surgery of 31 days for NAC-I and 34 days for NAC (p = 0.12). Delays to surgery after NAC have been correlated to worse survival outcomes, with delays greater than 8 weeks from completion of systemic treatment associated with worse survival [13, 14].

Notably, this study is one of only a few comparing surgical outcomes between patients with TNBC receiving NAC alone vs NAC-I. In this small retrospective study, patients in the NAC-I group had more advanced stage tumors and, though not statistically significant, numerically underwent more extensive surgery and reconstruction compared to patients in the NAC group. Nonetheless, there was no observed increase in PSCs in the NAC-I group with an average 90-day PSC rate of 35.2%. Most complications were considered minor including seroma requiring aspiration or cellulitis requiring antibiotics, however three patients in the NAC group experienced major complications requiring reoperation and/or hospitalization. Similarly, data from Memorial Sloan Kettering Cancer Center analyzing postoperative complications from 139 patients with undergoing NAC-I compared to 287 patients who received NAC alone showed no difference between the groups in terms of postoperative complications at 9.1% vs 7.9% (p = 0.6) [15]. These rates are notably lower than those of the current study, however rates and types of postmastectomy reconstruction were not reported to draw further conclusions.

In the current study, over half the patients underwent mastectomy and all but one patient undergoing mastectomy had some form of immediate reconstruction. Regardless of receipt of NAC, patients undergoing postmastectomy breast reconstruction will experience complications over 33% of the time [16, 17] and in the current study there was a trend toward increased PSCs in patients undergoing reconstruction on logistic regression (OR 0.20, CI 0.02–1.73, p = 0.721, Table 2). Furthermore, comparison of complications between autologous reconstruction to expander implant reconstruction have shown higher 2-year complication rates for autologous reconstruction but higher failure rate of expander implant reconstruction [16]. 24.1% of patients in this study underwent autologous reconstruction after NAC (n = 3) or NAC-I (n = 4), and while there was one patient with flap necrosis in the NAC group, autologous reconstruction was successful for all patients undergoing NAC-I.

Further studies have evaluated the impact of NAC on PSC rate. A recent meta-analysis by Loerntzen, et al. included 26 studies and 134,191 patients showed no increase in overall complications in patients receiving NAC [18]. Similarly, utilizing the American College of Surgeons National Surgical Quality Improvement Program (NSQIP) database, a study from Decker et. al examined the impact of neoadjuvant chemotherapy on wound healing in 44,533 patients and found no increase in wound complications from NAC [19]. However, this NSQIP study did observe a trend toward increased complications in NAC patients undergoing mastectomy with immediate reconstruction (OR, 1.58; 95% CI, 0.98–2.58).

A single-arm retrospective study out of MD Anderson also reported on perioperative complications in breast surgery after neoadjuvant immunotherapy [11]. In this study of 87 patients with TNBC treated with NAC-I, PSC rate was reported at 24.1%. Similar to our study, over 50% of patients in the MD Anderson study underwent mastectomy. However, in the cohort of patients in the current study, more patients underwent postmastectomy reconstruction, and almost one-quarter of patients who had reconstruction had autologous reconstruction, whereas only one patient in the MD Anderson cohort had autologous reconstruction. This difference in rate and type of reconstruction may account for some differences observed in complication rates.

PSCs are important when considering timing to adjuvant therapy. Evaluating a 90-day PSC window, rather than only a 30-day rate, can be useful in understanding why delays in therapy may occur as a late complication, which can ultimately have an impact on initiation of adjuvant treatment. Just as surgical delays can potentially impact survival [13], delays from surgery to adjuvant therapy have also been correlated with worse survival outcomes [20]. In the current study, median time to complications was 15 days (range 1–88) and patients in the NAC-I group had later complications compared to NAC patients (median 28 days vs 8.5 days, p = 0.034). While there were no major complications observed in the NAC-I group as opposed to 3 major complications seen in the NAC-group, the overall rate of complications between the groups was not significantly different and the time to adjuvant therapy was not worse for the NAC-I group. More importantly, the higher rate of pCR in the NAC-I group and the fewer patients in this group needing adjuvant chemotherapy likely contributed to the observed lower time to adjuvant therapy from surgery.

### Limitations

While this is one of a few studies only to compare PSCs between NAC and NAC-I, the relatively small number of patients included limits generalization. In this retrospective, exploratory study, baseline parameters between the groups were different and additional confounders including type and duration of neoadjuvant chemotherapy were not controlled for which could potentially impact outcome. Given the small number of patients undergoing reconstruction (n = 29), safety of reconstructive options after NAC-I will need to be validated in larger studies. Additionally, the patient population seeking care at UCLA is a relatively healthy population with higher health literacy, which may have influenced the lower number of comorbidities and complications. Finally, we acknowledge that the access to care and response to immunotherapy differ significantly when applying this study broadly, especially in minority patients receiving neoadjuvant immunotherapy who can experience overall worse post-surgical outcomes [21].

## Conclusion

In this study, we found that patients with TNBC receiving NAC-I have higher pCR rates and do not appear to have added 90-day PSCs compared to patients receiving NAC alone. Patients receiving NAC-I were also able to have successful immediate breast reconstruction, including autologous reconstruction. Fewer patients in NAC-I required adjuvant chemotherapy leading to a relatively shorter time to adjuvant treatments for this group. Larger prospective studies with matched patient groups will be useful to further understand surgical risk and optimize surgical outcomes after neoadjuvant immunotherapy.

## Data Availability

The data that support the findings of this study are not openly available due to reasons of sensitivity and are available from the corresponding author upon reasonable request. Data are located in controlled access data storage at the University of California Los Angeles.
